# Rectal wall dose-volume effect of pre- or post *KUSHEN Ningjiaos* relationship with 3D brachytherapy in cervical cancer patients

**DOI:** 10.1186/s13014-019-1354-5

**Published:** 2019-08-20

**Authors:** Xiaojuan Li, Cheng Xiao, Yilin Kong, Weiwei Guo, Wenting Zhan, Gong Li, Xuetao Wang, Bailin Zhang, Lei Gao

**Affiliations:** 1 Graduate student of grade 2016, Guangzhou University of Chinese Medicine, No.232, Waihuandong Road, University Town, Panyu District, Guangzhou, Guangdong China; 2 Radiation Oncology Department, Guangzhou University of Chinese Medicine Second Affiliated Hospital (Guangdong Provincial Hosiptal of Chinese Medicine), No. 55, Neihuanxi Road, University Town, Panyu District, Guangzhou, Guangdong China; 3 Radiation Therapy Department, Guangzhou University of Chinese Medicine Second Affiliated Hospital (Guangdong Provincial Hosiptal of Chinese Medicine), No.55, Neihuanxi Road, University Town, Panyu District, Guangzhou, Guangdong China

**Keywords:** Brachytherapy, Uterine cervical neoplasms, Matrine, Rectum, Cervical cancer

## Abstract

**Background:**

The present prospective study evaluated the safety and efficacy of the rectum following *KUSHEN Ningjiaos* in cervical cancer. We compared rectal wall changes during brachytherapy with or without *KUSHEN Ningjiaos* in cervical cancer patients and analyzed the difference in spatial dose distribution, including whole rectum-wall (R-w), anterior rectum-wall (R-a) and posterior rectum-wall (R-p).

**Methods and materials:**

One hundred cervical cancer patients with and without *KUSHEN Ningjiaos* were treated with brachytherapy (600 cGy). The whole R-w was divided into two areas of R-a and R-p, and R-w dose surface map were constructed. The volume of each R-w was compared in patients pre- and post-*KUSHEN Ningjiaos*.

**Results:**

When the pre- vs. post-*KUSHEN* groups were compared the volume of R-w increased. In the post-*KUSHEN* group, a significantly higher proportion of the D2cc of V_R-w_ and V_R-a_ compared with the pre-*KUSHEN* group showed that the D2cc_mean_ increased from 532.45 cGy to 564.7 cGy and 533.51 cGy to 565.26 cGy, respectively; however, results demonstrated a decrease in the D2cc_mean_ of R-p from 260.5 cGy to 240.0868 cGy (*P* < 0.05). The insertion of *KUSHEN Ningjiaos* resulted in a reduction of the relative volume of R-p exposed to high doses, and regressive analysis showed that the D_R-p_-max correlated most strongly with V_R-w_ and D2cc_R-p_ (*P* < 0.01 and *P* < 0.05, respectively).

**Conclusion:**

The insertion of *KUSHEN Ningjiaos* can protect the rectum. *KUSHEN Ningjiaos* appears to be safe and well tolerated; therefore, we believe that there will be fewer adverse events after brachytherapy for patients.

**Trial registration:**

A multi-center, prospective clinical trial for *KUSHEN Ningjiaos* was inserted into rectum to reduce the rate of radiation proctitis in three-dimensional brachytherapy of cervical cancer. ChiCTR1900021631. 2 Mar 2019-Retrospectively registered.

## Background

Cervical cancer is one of the most common cancers in women. It is a life-threatening health problem, accounting for the death of nearly 270,000 women each year worldwide [1]. Radical hysterectomy or radiotherapy, concurrent with chemoradiotherapy are recognized as effective treatment options for cervical cancer patients. Radiation therapy (RT) is one of the major treatment modalities, which includes external beam radiation therapy (EBRT) and brachytherapy. Although cervical cancer has an excellent tumor control rate and favorable prognosis, in developing countries, it can still lead to higher mortality, especially at the stage of III and IV.

Three-dimensional (3D) techniques are widely available that allows the delivery of high does of radiotherapy to the clinical target volume (CTV) while sparing the surrounding normal tissue, such as bladder and rectum, resulting in favorable toxicity profiles as compared to conventional radiotherapy. However, radioactive damage is a common side effect of radiotherapy and can occur even years after treatment, being a feared complication of radiotherapy in the rectum. Radiation proctitis (RP) is a serious late side effect after treatment. Radiation techniques to prevent RP are constantly improving thanks to image-guided and intensity-modulated radiotherapy. Therefore, how to decrease the radiation dose to the rectum and how to improve the quality of life in patients with cervical cancer who are undergoing brachytherapy are important questions to address.

Although the pathogenesis of RP remains unknown; it is known that firstly, mucosal damage is observed after radiation, subsequently, connective tissue is expanded and remodeled, and lastly, fibrosis and ischemia are observed [2]. A strong correlation has been observed between the presence and severity of telangiectases and the radiation dose administered to the rectal wall (R-wall) mucosa [3, 4]. The different dilatation of the R-wall caused a shift in the ratio between high-dose and low-dose exposed surface areas that might have a positive influence on mucosal regeneration [5–7]. Therefore, we propose a hypothetical theory that the rectal mucosa is a process of continuous repair. The mucosa of the posterior R-wall should be avoided as much as possible. Following radiotherapy, the rectal mucosa of the anterior R-wall is replaced by minor injury repair or undamaged posterior R-wall mucosa, and that continuous orientation growth and renewal reduces the incidence of radiation proctitis.

*KUSHEN Ningjiaos* is mainly composed of a mixture of matrine and carbopol. Matrine (C15H24N2O) is an alkaloid from *Sophora flavescens*, which is a traditional Chinese herb medicine, that has been identified to exhibit pharmacological effect, including anti-inflammatory [8], and anti-bacterial [9] effects, and exhibits multiple protective effects on cancers [10–13]. Gels are semisolid preparations that have the properties of the extract and the appropriate amount of matrix.

Some treatments have been created to overcome this issue of toxicity, according to the relationship between the delivered dose distribution to the rectum and the risk of late rectal toxicity in pelvic radiotherapy, which are proved to protect the focus on R-wall dose and the volume of the irradiated rectum [14–18]. In prostate cancer, increasing the prostate-rectum distance displaces the rectal wall away from the prostate and out of the regions of high-dose RT. [5–7, 17] The overall effect is a reduction in the maximum dose to the rectum and the total volume of irradiated rectum. However, there is no study in cervical cancer, therefore we hypothesize that creating space in the rectum will increase the distance from CTV to posterior rectum-wall (R-p) protecting the rectum. Thus, a prospective, non-randomized, open-label study in cervical cancer patients was carried out in our department.

## Methods and materials

We are describing a novel approach to decrease rectal toxicity and improve quality of life for patients with cervical carcinoma treated by brachytherapy. Transanal *KUSHEN Ningjiaos* inserted into the rectum was used to consistently push the rectal wall away from the high dose sources of radiation.

### Patients

A total of 100 patients indicated for a course of brachytherapy as a result of pathologically confirmed cervical cancer (FIGO: a stage of I-IV) that had undergone EBRT prior to brachytherapy were include in our study. All patients did not undergo a hysterectomy and ranged from 18 to 80 years old. If the patient was treated during the fertile window, she took contraceptive measures during the study. Patients were enrolled in the present study between 10 Apr 2018 and 31 Oct 2018. The patients received brachytherapy at Guangzhou University of Chinese Medicine Second Affiliated Hospital (Guangdong Provincial Hospital of Chinese Medicine). All patients gave written informed consent, according to the Helsinki Declaration. The study was approved by the local ethics review boards. Patients were excluded from the study if they had a combination of other gynecologic malignancies or other personal histories of non-gynecologic malignancies.

### Treatment

Patients with cervical cancer were enrolled in the clinical trial. The pre-*KUSHEN* patient group did not receive insertion of *KUSHEN Ningjiaos.* The post-*KUSHEN* patient group received insertion of *KUSHEN Ningjiaos*. We inserted 20 g (40 ml) of *KUSHEN Ningjiaos* into the rectum to increase the volume of the rectum and the distance between the CVT and the posterior rectal wall (R-p).

All patients, who had completed EBRT, were treated with high-dose-rate intracavitary brachytherapy (HDR-ICBT) using a remote after-loading system (microSelectron, Nucletron, the Netherlands) that employed an ^192^Ir source intraoperative planning with real-time dynamic dose calculation.

### Methods

The insertion of *KUSHEN Ningjiaos* in the rectum occurred after the implantation of all needles according to the following procedure.

Step 1: The patient was placed in the lithotomy position, and gynecologic examination was performed to assess the dimensions of the tumor, the degree of tumor extension. A sterile speculum was inserted into the vagina to expose the vagina and tumor. A single stainless steel tube applicator was inserted into the uterine cavity. Subsequently, stainless steel needles were inserted into the tumor. The location and the depth were determined by the gynecologic examination and CT/MRI. Tube and needles were fixed using a button stopper, and the vagina was packed with gauze to prevent needle movement. Before treatment begin, computed tomography (CT) simulation scans were performed in all patients. Each patient was scanned in the treatment position (supine) at 5-mm slice thickness.

**Fig. 1:**
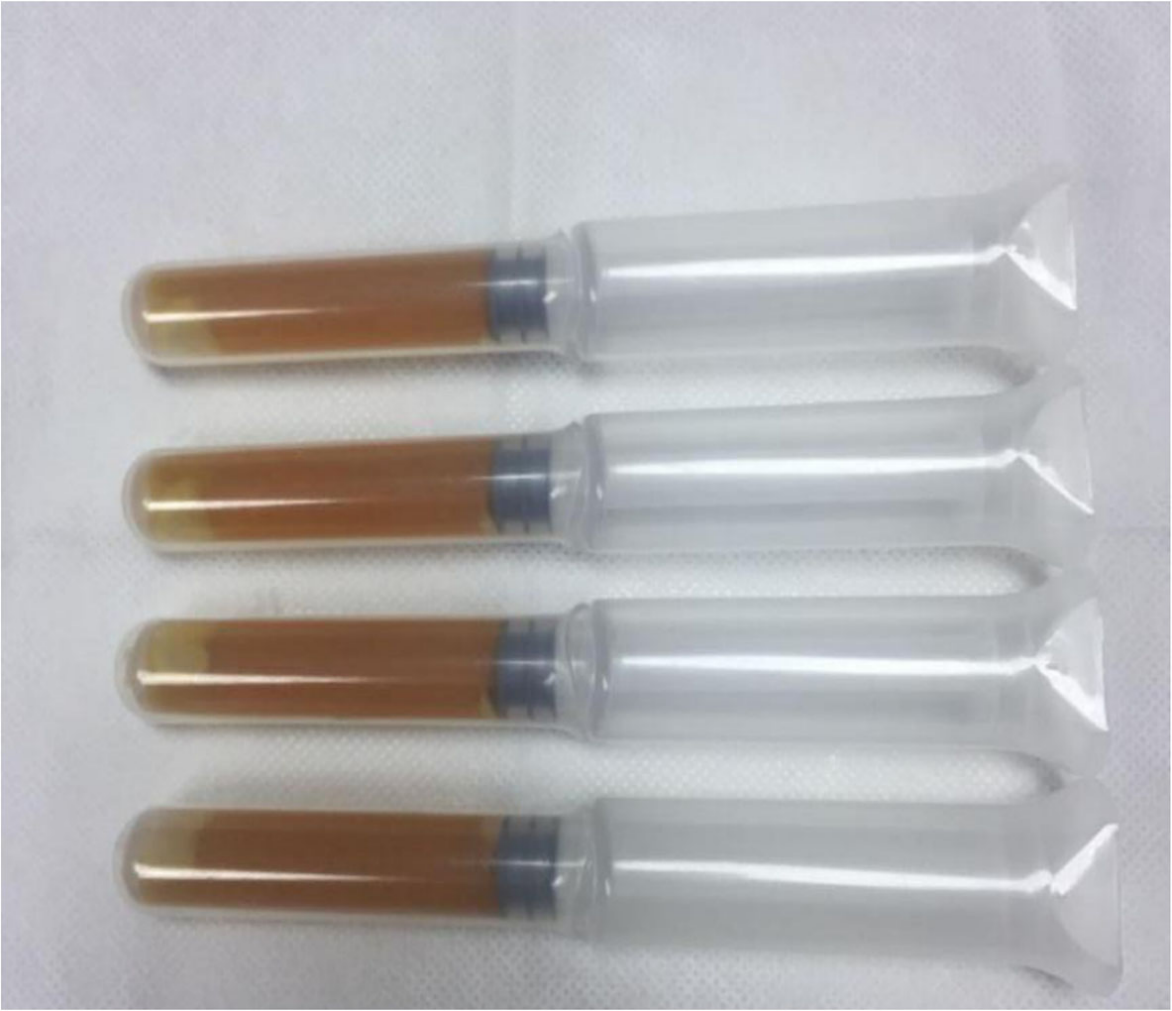
The KUSHEN Ningjiaos [20 g (5 g × 4)]

Step 2: After a baseline CT planning scan was complete, each patient was placed in the lithotomy position and *KUSHEN Ningjiaos* [20 g (5 g × 4), 10 cm length (Fig. 1)] were inserted into the rectum by the same attending radiation physician. Then the simulation scans were repeated, and the order of CT scanning was the same. The status of the *KUSHEN Ningjiaos* insertion was incorporated into the post-*KUSHEN* group.

**Fig. 2:**
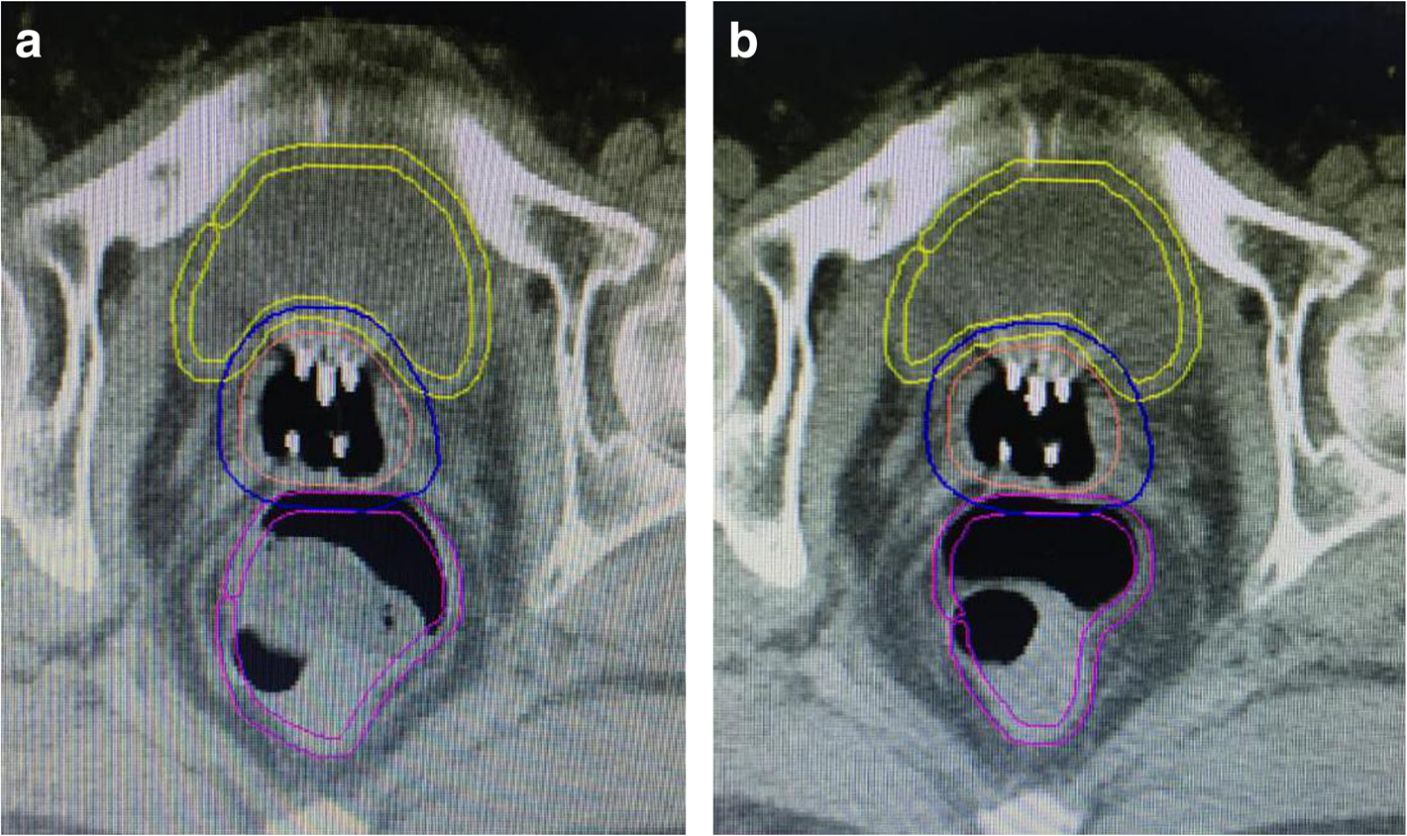
The CT images of a patient with KUSHEN Ningjiaos before insertion (**a**) and after insertion (**b**)

Step3: The images for all CT scans were imported into Oncentra treatment planning system, then patients were assigned to receive treatment with (post*-KUSHEN* group) or without *KUSHEN Ningjiaos* (pre-*KUSHEN* group) (Fig. 2) a.

Step 4: The same physician used PLATO Patient Selection System to decide the distribution of the treatment dose.

### Brachytherapy

Brachytherapy treatment planning was performed on simulation scans acquired before and after insertion of *KUSHEN Ningjiaos*. The treatment plans (including CT scan, delineated structures, and dose distributions) and the 3D-ICBT techniques were used.

Two planning computed tomography scans were performed, one without *KUSHEN Ningjiaos* and the other with *KUSHEN Ningjiaos*. In general, for the first and the second brachytherapy, CTV would include the uterus plus vagina. And the next three brachytherapy, we will delineate the CTV according to the different clinical condition of the patient by CT/MRI, which obtained the prescription dose of 600 cGy, and both expanded with a 5 mm 3D margin. On each slice, the bladder and rectal wall surrounding normal structures were outlined. Then the rectum wall was delineated from the ischial tuberosities up to the rectosigmoid flexure; both the anterior and posterior rectum-wall contours were outlined. After the definitive treatment plan was made, in all treatment plans, the volume of CTV, rectum-wall (including anterior wall, posterior wall, and whole wall), and bladder normal tissue were exposed to 600 cGy (V6), 500 cGy (V5), 400 cGy (V4), 300 cGy (V3), and 200 cGy (V2) were calculated. For spatial dose distribution analysis, dose surface histograms (DVHs) were generated.

### Statistical analysis

For the statistical analysis, the Stata package was used (release 14, Stata Corporation, College Station, TX). Sample proportions, means, and median values were used to describe the rectal wall characteristics. The T test was used for comparing proportions across groups. The Regressive Analysis test was used to compare mutual influence of the different measured quantities (V_R-w_, V_R-a_, V_R-p_, D2cc, D_R-p_-max) in the same subjects with and without *KUSHEN Ningjiaos*. A two-tailed *P* value less than 0.05 was considered statistically significant.

## Results

The insertion of *KUSHEN Ningjiaos* is a safe and simple procedure and was performed successfully in all patients. Most patients tolerated the insertion of *KUSHEN Ningjiaos* well, there are no unanticipated adverse events associated with the *KUSHEN Ningjiaos* procedure or the *KUSHEN Ningjiaos*.

### The volume of rectal wall (R-wall)

**Fig. 3:**
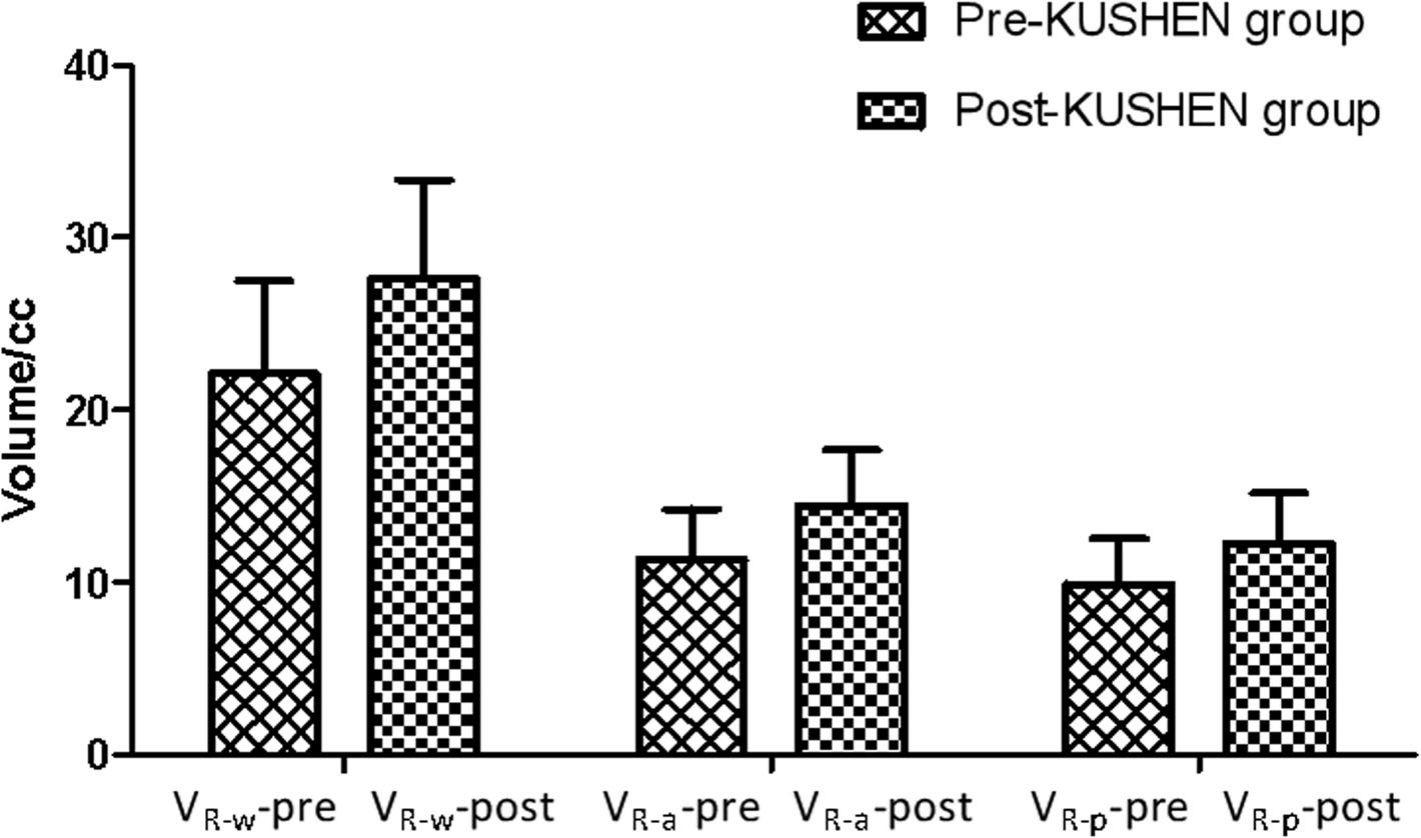
The mean volume of the R-wall with and without KUSHEN Ningjiaos.Abbreviations: these values represent mean ± SD. V_R-w_-pre = the volume of a whole R-wall without *KUSHEN Ningjiaos;* V_R-w-_-post = the volume of a whole R-wall with *KUSHEN Ningjiaos;* V_R-a_-pre = the volume of anterior R-wall without *KUSHEN Ningjiaos;* V_R-a-_-post = the volume of anterior R-wall with *KUSHEN Ningjiaos;* V_R-p_-pre = the volume of posterior R-wall without *KUSHEN Ningjiaos;* V_R-p_-post = the volume of posterior R-wall with *KUSHEN Ningjiaos*

We first analyzed the impact of the R-wall volume from the insertion of the *KUSHEN Ningjiaos*. The mean volume of the whole R-wall (± 1 SD) of the inserted *KUSHEN Ningjiaos* was 22.12 cc (± 5.38 cc) in the pre-*KUSHEN* group, 27.59 cc (± 5.78 cc) in the post-*KUSHEN* group. Insertion of the *KUSHEN Ningjiaos* resulted in an increase of the whole R-wall volume (R-wall encompasses anterior R-wall plus posterior R-wall). Without the *KUSHEN Ningjiaos*, the mean anterior R-wall (± 1 SD) was 11.33 cc (± 2.91 cc), when the *KUSHEN Ningjiaos* were inserted the anterior R-wall increased to 14.45 cc (± 3.22 cc). The mean volume of the posterior R-wall was 9.92 cc and 12.28 cc for the pre-*KUSHEN* and post-*KUSHEN* groups*,* respectively. The post-*KUSHEN* group thus produced 2.36 cc more than the pre-*KUSHEN* group. These differences were statistically significant (*P < 0.05*) and are shown in Fig*.* 3.

### The maximum dose of the posterior R-wall

**Fig. 4:**
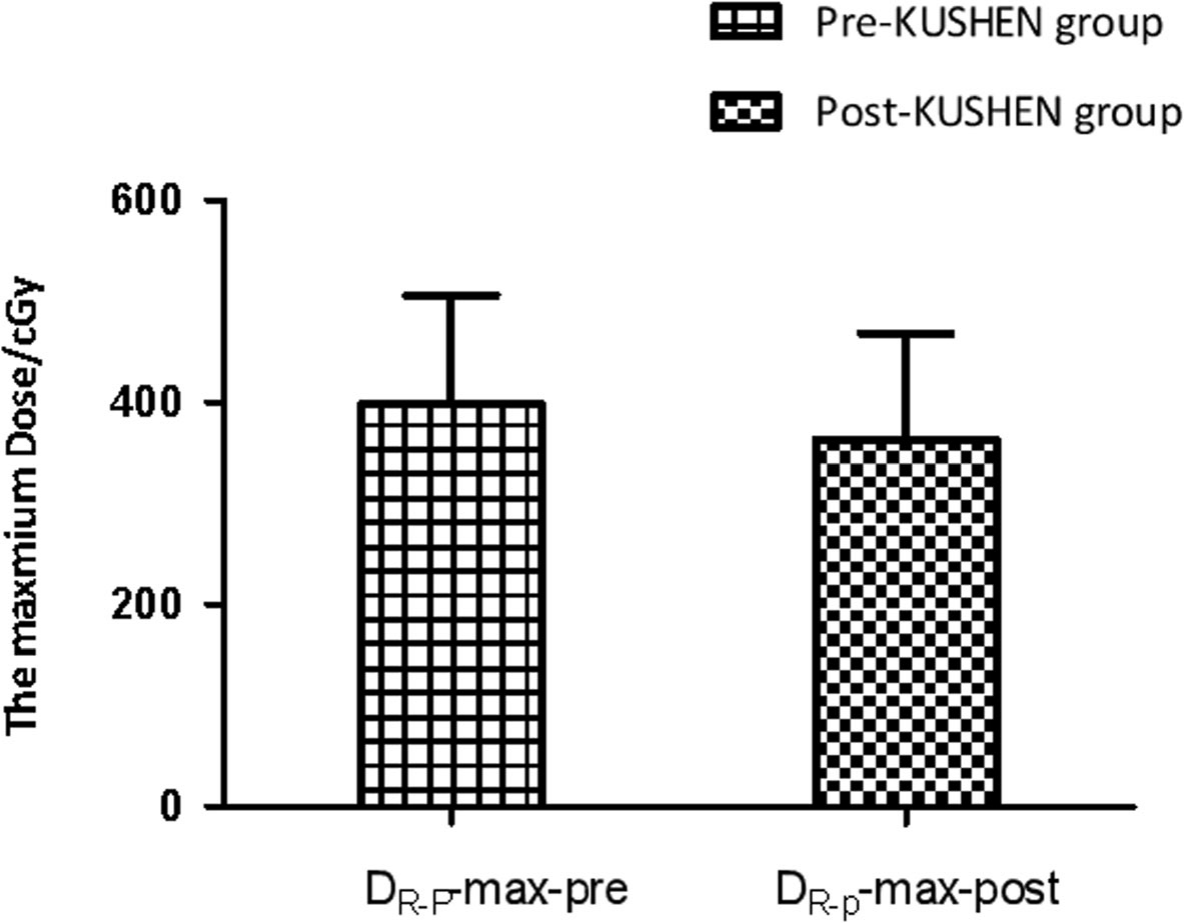
The maximum dose of the posterior R-wall with and without KUSHEN Ningjiaos.Abbreviations: D_R-p_-max-pre = the maximum dose of the posterior R-wall without *KUSHEN Ningjiaos;* D_R-p_-max-post = the maximum dose of the posterior R-wall with *KUSHEN Ningjiaos*

We next aimed to determine whether changes in the maximum dose of the posterior R-wall (D_R-p_-max) induced by insertion of *KUSHEN Ningjiaos* was associated with changes in the volume of the posterior R-wall (V_R-p_). As displayed in Fig. 4, The mean maximum dose of the D_R-p_-max was significantly reduced by using *KUSHEN Ningjiaos.* The mean D_R-p_-max post-*KUSHEN* group was 362.49 cGy (ranging from 138 to 594 cGy) and in the pre-*KUSHEN* group was 397.91 cGy (ranging from 156 to 594 cGy) and the difference was statistically significant (*P < 0.05*).

### The D2cc of R-wall

**Fig. 5:**
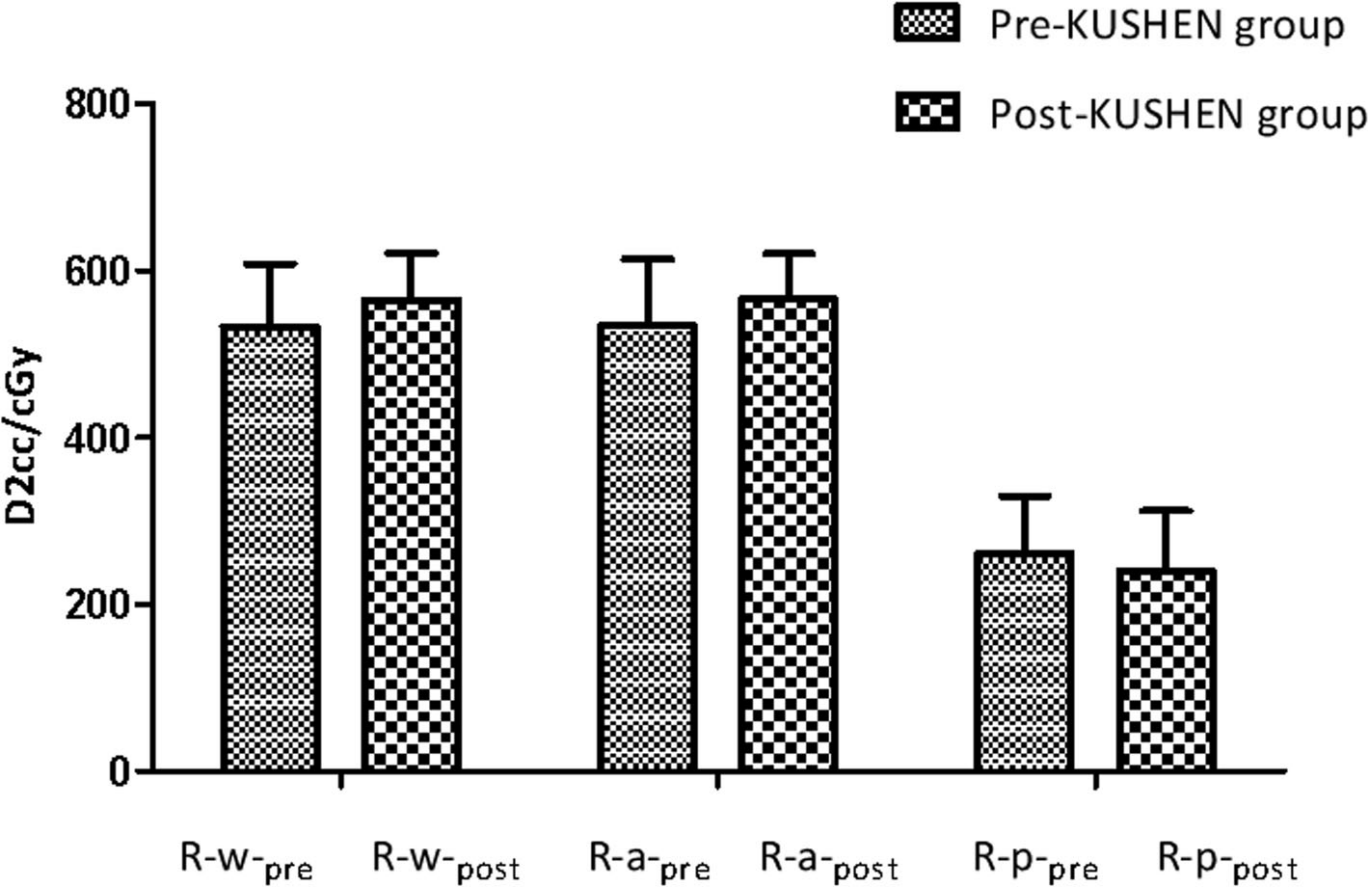
The D2cc of R-wall without and with KUSHEN Ningjiaos.Abbreviations: these values represent mean ± SD, D2cc_R-w_-pre = the D2cc of whole R-wall without *KUSHEN Ningjiaos;* D2cc_R-w_-post = the D2cc of a whole R-wall with *KUSHEN Ningjiaos;* D2cc_R-a_-pre = the D2cc of anterior R-wall without *KUSHEN Ningjiaos;* D2cc_R-a_-post = the D2cc of anterior R-wall with *KUSHEN Ningjiaos;* D2cc_R-p_-pre = the D2cc of posterior R-wall without *KUSHEN Ningjiaos;* D2cc_R-p_-post = the D2cc of posterior R-wall with *KUSHEN Ningjiaos*

In the post-*KUSHEN* group, the D2cc of R-wall in R-w and R-a increased compared with the pre-*KUSHEN* group (Fig. 5) and these differences were statistically significant (*P < 0.05*). The post-*KUSHEN* group demonstrated a higher proportion of D2cc in the whole R-wall compared with the pre-*KUSHEN* group [532.45 cGy (±75.71 cGy) vs. 564.7 cGy (± 56.00 cGy)]. For the anterior R-wall, the pre-*KUSHEN* group was 533.51 cGy (± 80.02 cGy) and the post -*KUSHEN* group was 565.26 cGy (± 54.72 cGy). However, the D2cc of R-p was 260.50 cGy (± 69.37 cGy) but after insertion of *KUSHEN Ningjiaos* decreased to 240.09 cGy (± 71.92 cGy) (*P < 0.05*).

### The maximum distance from posterior R-wall to CTV

**Fig. 6:**
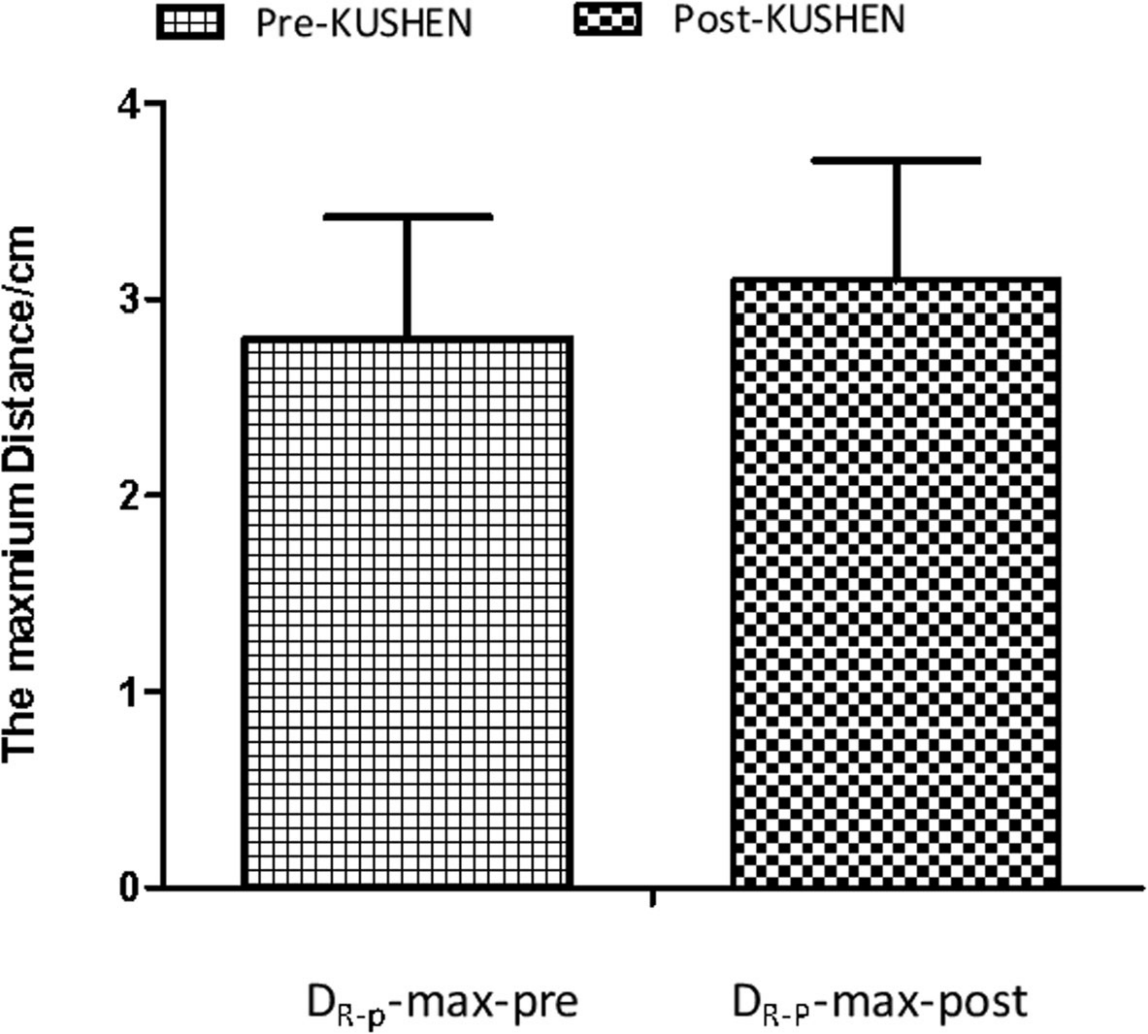
The maximum distance from posterior R-wall to CTV.Abbreviations: D_R-p_-max-pre = the maximum of distance from posterior R-wall to CTV without *KUSHEN Ningjiaos;* D_R-p_-max-post = the maximum of distances from posterior R-wall to CTV with *KUSHEN Ningjiaos*

The mean maximum distance from the posterior R-wall to CTV are displayed in Fig. 6. For the pre-*KUSHEN* group, the D_R-p_-max was 2.80 cm (± 0.62 cm), and the maximum distance from the posterior R-wall to CTV was higher in the post-*KUSHEN* group [D_R-p_-max = 3.10 cm (± 0.61 cm)] (*P < 0.05*).

**Fig. 7:**
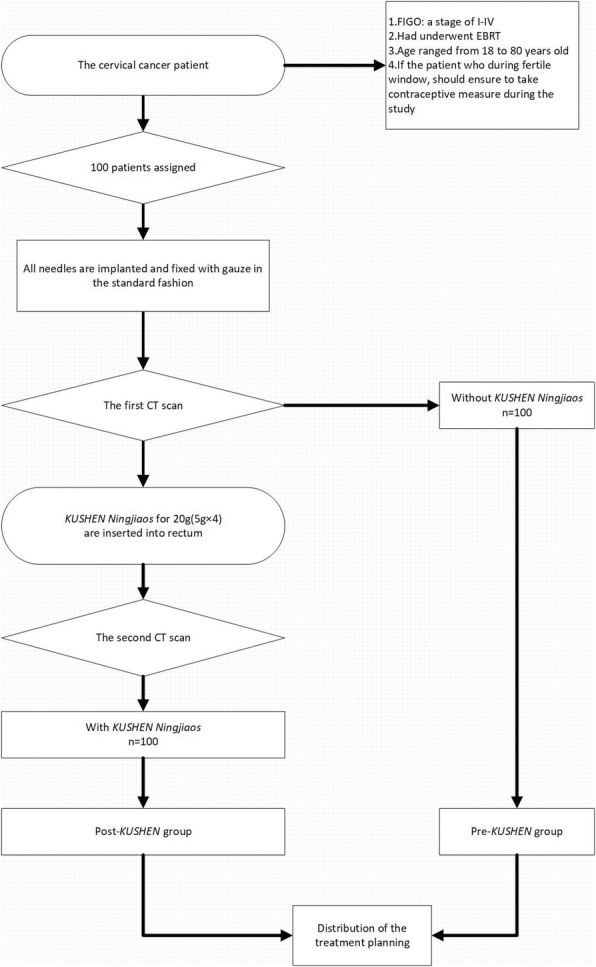
Depicts the flow chart of the methods used

### Potential factors affecting maximum dose of posterior R-wall

*KUSHEN Ningjiaos* resulted in a reduction of the relative volume of R-p exposed to high doses. A multivariate analysis was performed to determine potential factors that affect the maximum dose of posterior R-wall. The following parameters were used: the volume of R-wall, including whole, anterior and posterior R-wall; the distances from posterior R-wall to CTV; and the D2cc of the rectum-wall. Table 1 shows the results of the logistic regression analysis. The volume of anterior and posterior R-wall and the distance from the posterior R-wall to CTV were not correlated with the maximum dose of the posterior R-wall. Both the volume of whole R-wall (V_R-t_) and the D2cc of posterior R-wall (D2cc_R-p_) were most influenced by the maximum dose of posterior R-wall (*P < 0.01* and *P < 0.05*, respectively).

**Table 1:** A multivariate analysis of potential factors affecting maximum dose of posterior R-wall

Variable	m2
V_R-w_	−7.2503547*
V_R-p_	5.9519155
V_R-a_	(omitted)
D_R-p-max_	−8.1957419
D2cc_R-w_	−.03246589
D2cc_R-p_	.51348355***
_cons	11.268261

*Abbreviations*: *V*_*R-w*_ the volume of a whole R-wall, *V*_*R-a*_ the volume of anterior R-wall, *V*_*R-p*_ the volume of the posterior R-wall, *D*_*R-p*_*-max* the maximum dose of the posterior R-wall, *D2cc*_*R-w*_ the D2cc of whole R-wall, *D2cc*_*R-p*_ the D2cc of posterior R-wall

Legend: V_R-a_ omitted because of collinearity, * *p* < .1; ** *p* < .05; *** *p* < .01

## Discussion

Nowadays, three-dimensional conformal radiotherapy (3D-CRT) is widely available and allows the delivery of high doses to the CTV while sparing the surrounding normal tissues, such as the bladder and rectum. It is known that the majority of CTV is located in the zone of the cervical and vagina, consequently, the anterior rectal wall will inevitably be in the high-dose region. Radiation proctitis is a major complication for patients with cervical cancer following radiotherapy. RP can result in late rectal bleeding, and it is estimated that the incidence is range from 2 to 20% [19, 20]. In addition, a severe reduction in quality of life is observed [17, 21–24]. In our prospective study, we aimed to determine a protection method for the development of radiation proctitis after irradiation, and evaluated the efficacy of *KUSHEN Ningjiaos.*

The management of radiation proctitis is extremely challenging as no recommended guidelines are available and a limited number of studies have been published involving the various treatment options. The risk of rectal toxicity depends on the volume of the rectum that receives a high dose of radiation [25]. Therefore, it is important to implement techniques that prevent high rectal volume doses. There are many ways to reduce the dose to the rectum during radiation treatment, which have been proven effective. Several investigators have evaluated different materials, injected transperineally, to create space in the rectum, such as an absorbable hydrogel or hyaluronic acid collagen injections [15, 17, 18]. Additionally, newer techniques, such as new devices like rectum spacers and balloons, have been developed to spare rectal structures [18, 26]. Although these materials have been shown to reduce the rectal toxicity, each has certain drawbacks, such as an absorbable hydrogel has a short persistence and a non-uniform distribution. Hyaluronic acid (HA) injections have potential risks; the implantation of rectum spacers is typically performed transperineally under real-time transrectal ultrasound guidance. The insertion procedure can be performed under local (with or without sedation), spinal, or general anesthesia [18]. Therefore, it is not conducive to use during treatment. Endorectal balloons are inserted into the rectum during treatment to increase the distance from the dorsal rectal wall to the CTV. The anterior rectal wall is pushed towards the CTV, however the overall effect proved to be beneficial in radiotherapy [17], but the balloon remains in the rectum during treatment and effected the comfort of the patient.

*KUSHEN Ningjiaos* is mainly composed of matrine. Matrine has an anti-tumor, anti-inflammatory, and antibacterial effect. It is absorbable, non-toxic, and non-immunogenic, and it can change according to the shape of rectum. *KUSHEN Ningjiaos* overcomes the small contact area of a suppository, is difficult to infiltrate the mucous membrane, and the action time of the enema liquid is short, which is unfavorable to the shortcomings of retention. With good bioadhesiveness, it is convenient for retention in the intestine and improves bioavailability in the rectal administration. *KUSHEN Ningjiaos* reduced the posterior R-wall surface that was exposed to intermediate and high doses of radiation by pushing the posterior rectum-wall away from the high-dose region, thus increasing the distance from the posterior rectal wall to the radioactive sources. Overall, the safety results show that the insertion of *KUSHEN Ningjiaos* appears to be well tolerated. The insertion procedure was found to be fast; the overall progress time from the insertion *KUSHEN Ningjiaos* to removal was 1 min. The present study had several limitations, inherent to its design, that is worth mentioning. As a new technique, the use of *KUSHEN Ningjiaos* is limited by clinical data and treatment modes. The procedure for *KUSHEN Ningjiaos* insertion could have some disadvantages. Potential side effects could occur, such as urgency and tenesmus, these risk factors are not yet fully described and are estimated to be very low.

The principal aim of our study was to reduce irradiation to the rectum, and understand the factors associated with reducing the rectum exposure to high dose areas. We attempted to minimize rectal toxicity by increasing the distance between the CTV and rectum and the volume of rectum. We demonstrated that the increase in the volume of whole R-wall and the D2cc of posterior R-wall created by the insertion of *KUSHEN Ningjiaos* provided a significant radiation dose reduction when patients were treated with brachytherapy. High dose area of the rectum has the crucial role in radiation proctitis. Our results showed that the insertion of *KUSHEN Ningjiaos* reduce the area of the rectum exposing to a high dose of radiation. Although evidence showed that usually the D2cc of the anterior rectal wall is predictive for rectal toxicity, as expected almost whole and anterior rectal wall dose parameters become worse after the insertion of *KUSHEN Ningjiaos*, but the posterior R-wall improved.

According to radiobiology, rectal mucosa is an early responding tissue with the characteristics of rapid cell renewal. After being damaged, the active proliferation is used to maintain the stability of the number of cells in the tissue and to promote the recovery of the damaged tissue. On the other hand, rectum has the characteristics of late effects tissue. Obliterative endarteritis, chronic mucosal ischemia, submucosa fibrosis and new vessel formation are acknowledged as pathologic reasons for CRP [27]. Although this point has gradually been taken seriously [28], no relevant research has been found.So we try to reduce the D2cc of posterior rectal wall to protect enough posterior rectal wall.The cells of posterior rectal wall were protected, like the stem cell, they can proliferate and regenerate by enriched blood supply, and the cell population can crawl along the rectal wall, and then the necrotic or fibrotic cells were renewed and replaced, and the damaged cells of the anterior rectal wall are repaired.

According to the results of a multivariate analysis of potential factors that affected the maximum dose of posterior R-wall, other factors, including distance, are not associated. We postulate that the reason is that the shape of the rectum is irregular; therefore, after insertion of *KUSHEN Ningjiaos*, the distance does not increase enough to protect the rectum from high-dose radiation. However, air and stool amount will cause a different between the distance of the rectum. Small areas of rectal wall exposed to high dose are believed to regenerate faster if surrounded by areas of low dose [5, 6]. Thus, increasing the volume of the whole R-wall can reduce the area of the rectum exposed to a high dose of radiation.

Ended by Mar 2019,fifty seven patients had received our followed-up.Within a median of 7 months follow-up, each patient was assessed by imageological examination(CT/MRI) and/or clinically and/or endoscopically. Radiation proctitis was score using RTOG. The results showed acute radiation proctitis with 15/57 patients, acute gastro-intestinal(GI) toxicities were noted in 10/15 patients having grade 1, 5/15 with grade 2, no acute grade 3 and 4 toxicities were noted. Chronic radiation protitis with 22/57 patients, the results showed chronic GI toxicities with 19/22 patients having grade 1, and 3/22 patients with grade 2 chronic toxicities, no grade 3 and 4 chronic effects were reported. The incidence and the serious level of radiation proctitis were reduced.

Currently available data are limited by relatively short follow-up. Late effects of higher grade have been shown to occur at a median of 1.5 and 4.5 years, so longer follow-up is required to determine further possible effects of *KUSHEN Ningjiaos* on late toxicity [29].

## Conclusion

In conclusion, the use of *KUSHEN Ningjiaos* provided a reduction of radiation to the posterior rectal wall. *KUSHEN Ningjiaos* appears to be safe and well tolerated when administered appropriately. More clinical data and longer follow-up are necessary to define the precise role of the *KUSHEN Ningjiaos* in combination with radiation proctitis.

## Data Availability

All data analysed during this study are included in this published article. More clearly datas are available from the corresponding author on reasonable request.
